# Exploring Mucoadhesive and Toxicological Characteristics Following Modification of Linear Polyethylenimine with Various Anhydrides

**DOI:** 10.1021/acs.biomac.4c00220

**Published:** 2024-07-29

**Authors:** Manfei Fu, Roman V. Moiseev, Matthew Hyder, Wayne Hayes, Silvia Amadesi, Adrian C. Williams, Vitaliy V. Khutoryanskiy

**Affiliations:** † School of Pharmacy, University of Reading, Whiteknights, Post Office Box 224, Reading RG6 6DX, U.K.; ‡ Physicochemical, Ex Vivo and Invertebrate Tests and Analysis Centre (PEVITAC,www.pevitac.co.uk), University of Reading, Whiteknights, Reading RG6 6DX, U.K.; § Department of Chemistry, University of Reading, Whiteknights, Post Office Box 224, Reading RG6 6DX, U.K.

## Abstract

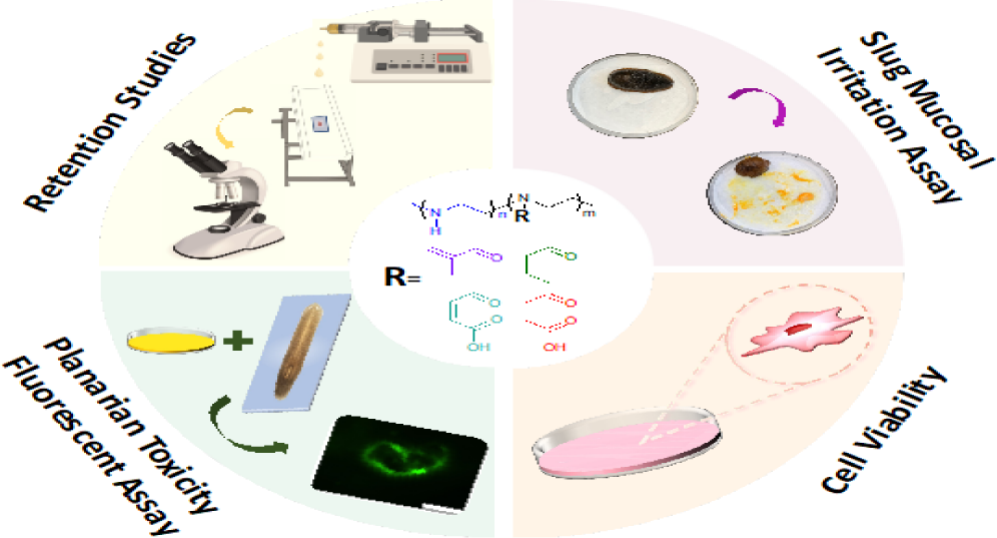

Linear polyethylenimine (L-PEI) has numerous applications, such as in pharmaceutical formulations, gene delivery, and water treatment. However, due to the presence of secondary amine groups, L-PEI shows a relatively high toxicity and low biocompatibility. Here, various organic anhydrides were used to modify L-PEI to reduce its toxicity and enhance its functionality. We selected methacrylic anhydride, crotonic anhydride, maleic anhydride, and succinic anhydride to modify L-PEI. The structure of the resulting derivatives was characterized using ^1^H NMR and FTIR spectroscopies, and their behavior in aqueous solutions was studied using turbidimetric and electrophoretic mobility measurements over a broad range of pHs. A fluorescence flow through method determined the mucoadhesive properties of the polymers to the bovine palpebral conjunctiva. Methacrylated L-PEI and crotonylated L-PEI showed strong mucoadhesive properties at pH 7.4, likely due to covalent bonding with mucin thiol groups. In contrast, maleylated and succinylated L-PEI were poorly mucoadhesive as the pH was above their isoelectric point, resulting in electrostatic repulsion between the polymers and mucin. The toxicity of these polymers was evaluated using in vivo assays with planaria and the 3-(4,5-dimethylthiazol-2-yl)-2,5-diphenyl-2*H*-tetrazolium bromide (MTT) cell viability assay in human alveolar epithelial cells. Moreover, the irritancy of polymers was assessed using a slug mucosa irritation assay. The results demonstrated that anhydride modification mitigated the adverse toxicity effects seen for parent L-PEI.

## 1 Introduction

Mucus is a biological barrier covering epithelial cells of the respiratory system, reproductive system, and gastrointestinal tract^1^ to protect the underlying membranes.^2^ Mucin is a primary component of mucus^3^ and comprises a glycoprotein backbone and primarily O-linked glycan structures arranged in a bottlebrush-like conformation.^4^ Mucoadhesive polymers are commonly classified as anionic-, cationic-, amphoteric, or neutral polymers; chitosan, xanthan gum, and proteins are examples of charged polymers and exhibit relatively strong mucoadhesive properties.^5^ Electrostatic interactions are usually predominantly responsible for mucoadhesion while hydrogen bonding and hydrophobic effects can also contribute.^6^ Mucoadhesion has been extensively used in drug delivery to enhance the retention time of formulations, employing chitosan,^7^ xanthan gum,^8^ and weakly cross-linked poly(acrylic acid).^9^

Linear poly(ethylenimine) (L-PEI) has been explored for various applications,^10^ such as gene delivery,^11−13^ water purification,^14^ to produce functional inorganic minerals,^15^ and in PEI conjugates.^16−18^ Due to the presence of cationic secondary amine groups within L-PEI, which can interact with negatively charged mucin, L-PEI has also gained some attention for mucoadhesive applications in nasal^19^ and buccal drug delivery.^20^

Despite the numerous studies employing L-PEI as a gene delivery vector or as a pharmaceutical excipient, it is cytotoxic,^21^ predominantly attributed to electrostatic interactions with cell membranes and the extracellular matrix.^22^ Additionally, different structures, molecular weights, and macromolecular flexibility have been correlated with toxicity and delivery efficiency of L-PEI.^23^

Previously, Bianco-Peled and colleagues^24−26^ pioneered a method to enhance the mucoadhesive properties of various polymers—both cationic, anionic, as well as neutral materials such as alginate, chitosan, and Pluronic F127—by conjugating them with unsaturated acryloyl groups. The enhanced mucoadhesive ability of these polymers was attributed to a Michael addition click reaction occurring between the acryloyl groups within the mucoadhesive polymer and the thiol groups present in mucins under physiologically relevant conditions. In an NMR spectroscopic study, they reported the disappearance of vinyl protons of polyethylene glycol diacrylate when mixed with mucin.^27^ Subsequently, our research group demonstrated a similar enhancement in the mucoadhesive properties of cationic, anionic, and neutral polymers upon the introduction of methacryloyl moieties.^28^ In our most recent study,^29−31^ we further showed that the mucoadhesive properties of gelatin could be significantly enhanced by modifying this biopolymer through reactions with crotonic, itaconic, and methacrylic anhydrides. It was established that methacryloyl groups exhibit a superior ability to enhance mucoadhesive properties compared with crotonoyl and itaconoyl groups.

In this study, we further investigated the impact of introducing functional groups into water-soluble polymers, with the aim to enhance their mucoadhesive properties. This time, a series of amphoteric and cationic polymers were synthesized by modifying L-PEI with methacrylic anhydride, crotonic anhydride, maleic anhydride, and succinic anhydride. These new polymers were fully characterized by ^1^H NMR and FTIR spectroscopies. A fluorescence flow through the *ex vivo* method was used to assess the retention of these polymers on bovine palpebral conjunctiva. The toxicity of the polymers was evaluated *in vivo* using the model planaria assay^32^ and slug mucosal irritation test^33^ and an *in vitro* 3-(4,5-dimethylthiazol-2-yl)-2,5-diphenyl-2*H*-tetrazolium bromide (MTT) cell viability assay in human alveolar epithelial cells. This study clarifies how the nature of unsaturated groups affects their ability to enhance the mucoadhesive properties of L-PEI.

## 2 Materials and Methods

### 2.1 Materials

Poly(2-ethyl-2-oxazoline) (PEOZ, MW ∼50 kDa, PDI 3–4), succinic anhydride, maleic anhydride, methacrylic anhydride, crotonic anhydride, dimethyl sulfoxide (DMSO), triethylamine (TEA), deuterium oxide (D_2_O), deuterium methanol (MeOD-d4), fluorescein isothiocyanate (FITC), fluorescein isothiocyanate-dextran (FITC-dextran, average MW 10 kDa), gelatin, branched polyethylenimine (b-PEI, average MW 25 kDa), fluorescein sodium salt, benzalkonium chloride (BAC), fetal bovine serum (FBS), Dulbecco’s phosphate buffered saline (DPBS), nutrient mixture F-12 ham, Hanks’ Balanced Salt Solution (HBSS), trypsin-EDTA solution (EDTA), 6-diamidino-2-phenylindole (DAPI), thiazolyl blue tetrazolium bromide (MTT), formaldehyde solution 4% buffered (pH 6.9), penicillin/streptomycin, and propidium iodide (PI) were obtained from Sigma-Aldrich (Gillingham, U.K.). Urea, hydrochloric acid (37%), sodium hydroxide, magnesium sulfate, magnesium chloride, potassium chloride, phosphate-buffered saline (PBS) tablets, sodium bicarbonate, sodium chloride, and calcium chloride dihydrate were obtained from Fisher Scientific (Loughborough, U.K.). A dialysis membrane (MWCO 3.5 kDa) was purchased from Medicell Membranes Ltd. (U.K.). All other chemicals were of analytical grade and used without further purification.

### 2.2 Synthesis of Linear Polyethylenimine

L-PEI was synthesized by acidic hydrolysis of PEOZ following the protocol of Shan *et al*.^34^ Briefly, PEOZ (5.0 g) was dissolved in 50 mL of 37 wt % HCl before 50 mL of deionized water was added and heated overnight at 100 °C. Then, the L-PEI solution was diluted in cold deionized water. Cool NaOH aqueous solution (4 M) was added dropwise to the L-PEI solution until the L-PEI precipitated at pH 10–11.^35^ The precipitate was washed with deionized water until neutral pH and dried in a vacuum oven to obtain L-PEI yielding 1.90 g (89%).

### 2.3 Synthesis of Methacrylated L-PEI, Crotonylated L-PEI, Maleylated L-PEI, and Succinylated L-PEI

Either methacrylic anhydride (1.5 eq, 5.4 g), or crotonic anhydride (1.5 eq, 5.4 g), or maleic anhydride (1.5 eq, 3.4 g), or succinic anhydride (1.5 eq, 3.5 g) were dissolved in 15 mL dimethyl sulfoxide (DMSO) and then mixed with 45 mL of L-PEI (1 eq, 1.0 g) in DMSO, before triethanolamine (1.5 eq, 3.3 mL) was added. The mixture was stirred overnight at 40 °C. The obtained polymer solution was diluted with deionized water and dialyzed against deionized water (MWCO = 3.5 kDa) for 72 h. All polymers were recovered by freeze-drying. The following product yields were recorded for methacrylated L-PEI, crotonylated L-PEI, maleylated L-PE, and succinylated L-PEI: 2.23 g (86%), 2.31 g (91%), 2.60 g (79%), and 2.08 g (89%), respectively.

### 2.4 Characterization of Methacrylated L-PEI, Crotonylated L-PEI, Maleylated L-PEI, and Succinylated L-PEI

#### 2.4.1 ^1^H-Nuclear Magnetic Resonance Spectroscopy

Ten milligram of methacrylated L-PEI, crotonylated L-PEI, maleylated L-PEI, or succinylated L-PEI was dissolved in 1 mL D_2_O, whereas L-PEI was dissolved in 1 mL methanol-d4. The samples were transferred to an NMR tube and analyzed with a Bruker spectrometer operating at 400 MHz. All chemical shifts are given in ppm. MestReNova software (version 9.1.0) was used for spectral analysis. The degree of substitution (DS) of methacrylated L-PEI, crotonylated L-PEI, maleylated L-PEI, and succinylated L-PEI was calculated using peak integration, according to eqs 1-4, respectively:
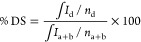
1
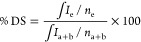
2
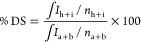
3
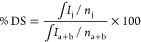
4where *I*_a_ is an integral of the signal assigned to the −CH_2_CH_2_– on the backbone of unreacted L-PEI, *I*_b_ is associated with −CH_2_– adjacent with the substituted nitrogen, *I*_d_ from the methacrylated L-PEI spectrum is attributed to methyl group of methacrylic anhydride, *I*_e_ of crotonylated L-PEI spectrum is attributed to methyl group of crotonic anhydride, *I*_h_ and *I*_i_ of maleylated L-PEI spectrum is attributed to methyne group of maleic anhydride, *I*_j_ of succinylated L-PEI spectrum is attributed to methenyl group of succinic anhydride.

#### 2.4.2 Fourier Transform Infrared Spectroscopy

Polymers were analyzed from 4000 to 950 cm^–1^ at a resolution of 4 cm^–1^ taking 64 scans using a diamond sampling accessory. Data were recorded by a Nicolet iS5 spectrometer (Thermo Scientific, U.K.) and plotted using OriginLab version 9.0 software.

#### 2.4.3 Gel Permeation Chromatography

GPC analysis was conducted on an Agilent Technologies 1260 Infinity II system, using HPLC grade DMF containing 5 mM NH_4_BF_4_ at a flow rate of 1 mL/min. Calibration was achieved using a series of near monodisperse PEO/PEG standards; samples were prepared at 1.0 mg/mL, with toluene used as a flow marker at 23.2 min.

#### 2.4.4 Turbidity Measurements

The effects of pH on solution turbidity of the modified L-PEIs were studied by using a JENWAY 7315 spectrophotometer (Bibby Scientific Ltd., U.K.). All samples were dissolved in deionized water (1 mg/mL) and turbidity recorded at 400 nm as pH was varied by adding 0.1 M NaOH or HCl. Each titration was repeated in triplicate, and the turbidity values are reported as mean ± standard deviation.

#### 2.4.5 Electrophoretic Mobility Measurements

The effects of pH on electrophoretic mobility of the polymers were studied in folded DTS-1070 capillary cells using a Malvern Zetasizer Nano-S instrument (Malvern Instruments, U.K.). All samples were dissolved in deionized water (1 mg/mL), and the pH was adjusted by adding 0.1 M NaOH or HCl. Measurements were conducted at 25 °C and repeated in triplicate; reported values are the mean ± standard deviation.

### 2.5 *Ex Vivo* Mucoadhesion Studies

#### 2.5.1 Preparation of Simulated Tear Fluid

Simulated tear fluid (STF) was prepared according to the protocol previously described by Moiseev *et al*.^36^ Briefly, 6.7 g NaCl, 2.0 g NaHCO_3_, and 0.8 g CaCl_2_·2H_2_O were dissolved in 1 L of deionized water and then adjusted to pH 7.40.^37^ STF was kept at 37 °C throughout the experimentation.

#### 2.5.2 Preparation of Fluorescently Labeled Polymers

Methacrylated L-PEI, crotonylated L-PEI, maleylated L-PEI, and succinylated L-PEI were labeled with FITC, according to our previously reported protocol.^38^ Polymer solutions (2 mg/mL) were prepared in 0.1 M carbonate buffer (pH 9), and FITC was dissolved in DMSO (1 mg/mL). The FITC solution was added to the polymer solutions at 1:20 v/v (FITC/polymer) and then incubated in a light proof container with overnight stirring at room temperature. The polymer–FITC solutions were dialyzed against 2.5 L of 0.01 M phosphate-buffered saline (PBS) using a cellulose membrane with MWCO 3.5 kDa at pH 7.4 for 72 h and then recovered by freeze-drying. Successful labeling of these polymers was confirmed using a fluorescence spectrophotometer (CARY Eclipse, US). The resultant polymers were dissolved in deionized water at 1 mg/mL. The excitation wavelength was 490 nm, and the emission wavelength range was 500–600 nm at room temperature (25 ± 3 °C). The emission and excitation slit widths were set at 5 nm, the emission voltage was 500 mV and the scan speed was 600 nm/min. Data were recorded and plotted using OriginLab version 9.0 software.

#### 2.5.3 Retention Studies on Ocular Tissues

Retention of FITC-labeled polymers on bovine palpebral conjunctiva was studied with FITC-dextran used as a negative control, following a modified protocol we previously reported.^39,40^ Bovine palpebral conjunctiva was dissected with a scalpel avoiding contact with surfaces. The ocular tissue (4 × 2 cm^2^) was mounted on a glass slide, with the mucosal side upward, and prerinsed with 1 mL freshly prepared STF. Briefly, the background fluorescence of the tissue (*I*_background_) was determined. Then, 40 μL of 1 mg/mL FITC-methacrylated L-PEI, FITC-crotonylated L-PEI, FITC-maleylated L-PEI, FITC-succinylated L-PEI, or FITC-dextran solution in STF was applied onto the mucosal surface, and fluorescence images were recorded to get initial fluorescence intensities (*I*_0_). After 3 min of dosing, the mucosal tissue was washed with STF using a syringe pump (Harvard Apparatus model 981074; Holliston, MA, US) at 0.1 mL/min, exceeding the normal human tear rate (1–2 μL/min).^40^ All experiments were conducted at 34.5 °C in an incubator.^41^ Fluorescence images of the mucosal tissue (*I*_t_) were acquired periodically using a Leica MZ10F stereomicroscope (Leica Microsystems, Wetzlar, Germany) with the GFP filter-fitted Leica DFC3000G digital camera at 3.2× magnification, 80 ms of exposure time, 2.0× gain, 1.0× gamma, and pseudo color at 520 nm. The acquired microscopy images from each time point were analyzed using ImageJ software (version 1.53t, 2022), and the fluorescence intensity was calculated according to eq 5:
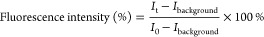
5where the zero-time point was set as 100%.

The results are presented as fluorescence intensity as a function of the time of irrigation (0–30 min) after the background fluorescence is subtracted from each image. Measurements were repeated in triplicate, and all values are reported as mean ± standard deviation.

### 2.6 Slug Mucosal Irritation Assay

#### Arion Lusitanicus

 slugs were collected locally (Reading, U.K.), housed in plastic containers at room temperature, and fed lettuce and carrots. The slug mucosal irritation test (SMIT) was conducted for methacrylated L-PEI, crotonylated L-PEI, maleylated L-PEI, and succinylated L-PEI according to a previously published protocol.^33^ To conduct experiments, slugs weighing 6–14 g, without macroscopic injuries and with clear tubercles and foot surfaces, were selected and housed separately in 1.5 L glass beakers. Twenty milliliters of PBS solution at pH 7.40 was used to soak a paper towel sheet in the base of each beaker and covered with cling film perforated with a needle, allowing air exchange. Slugs were maintained without food for 48 h at room temperature prior to experiments. On the day of the experiment, slugs were individually weighed and then placed in a 90 mm plastic Petri dish lined with Whatman filter paper soaked in 2 mL of 1.0 mg/mL of each polymer solution in PBS or 1% benzalkonium chloride (BAC) in PBS solution as a positive control or b-PEI as a second positive control or PBS solution alone as the negative control. Immediately following the 60-min contact period, the slugs were removed from the Petri dishes, rinsed with 10 mL of PBS solution, wiped gently with a paper towel, and reweighed. The amount of mucus produced (MP%) by each slug in response to the contact with the chemicals was calculated by
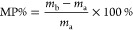
6where *m*_b_ and *m*_a_ are slug weights before and after exposure to test solutions, respectively. Tests used 5 slugs per solution, with data presented as the mean ± standard deviation.

### 2.7 Toxicology

#### 2.7.1 Acute Toxicity Assay

2.7.1.1

*Schmidtea Mediterranea* planaria were bred from a colony generously donated by Dr Jordi Solana (Oxford Brookes University). Planaria were maintained in an artificial pond water (APW) at 25 ± 3 °C in the dark, feeding calf liver twice per week. The APW was prepared with 3.2 mL 5 M NaCl, 10 mL 1 M CaCl_2_·6H_2_O, 10 mL 1 M MgSO_4_, 1 mL 1 M MgCl_2_, 1 mL 1 M KCl, and 1.008 g NaHCO_3_ in 10 L Milli-Q water and adjusted pH to 7–8 by adding 5 M HCl. The APW was changed every 3–4 days.

The planaria toxicity assay was modified from the method of Buang *et al*.^42^ Methacrylated L-PEI, crotonylated L-PEI, maleylated L-PEI, succinylated L-PEI, and b-PEI were dissolved at 1 mg/mL in the APW. Individual planaria were placed in 12-well culture plates and treated with 4 mL of each polymer solutions, or b-PEI (used as a positive control), or APW alone (used as a negative control). Planaria were treated for 1, 24, and 48 h. The number of live animals (with detectable movement) and dead animals (without detectable movement) was recorded. Five biological replicates were obtained for each of the treatments and for each time point.

#### 2.7.2 Planarian Toxicity Fluorescent Assay

The toxicity fluorescent assay was slightly modified from Shah *et al*.^32^ Planaria that remained viable following the acute toxicity assay were washed with APW for 1 min, exposed to 0.1% (w/v) sodium fluorescein solution in APW for 1 min, and then washed with APW for 1 min to remove excess sodium fluorescein. Planaria were then placed on a microscope glass slide and immobilized with a few drops of 12% (w/v) gelatin solution and placed on ice. Fluorescence images were collected using a Leica MZ10F stereomicroscope (Leica Microsystems, U.K.) fitted with a DFC3000G digital camera set at 970 ms exposure time, 2.0× magnification, 5.1× gain, 0.7× gamma, and pseudo color at 520 nm. The images were analyzed using ImageJ software (version 1.53t, 2022). Five replicates with different worms were taken for each treatment, and fluorescence intensity values are reported as the mean ± standard deviation.

#### 2.7.3 Cell Viability

##### 2.7.3.1 Cell Culture and Treatment

A549 cells were kindly provided by Prof Darius Widera (University of Reading, School of Pharmacy, Reading, U.K.). The cells were cultured in Ham’s F-12 Nutrient Mixture (F-12) supplemented with 10% fetal bovine serum (FBS) and 100 U/mL penicillin and 100 μg/mL streptomycin. For the treatments, the synthesized polymers and b-PEI were dissolved in F-12 supplemented with 1% FBS and 100 U/mL penicillin and 100 μg/mL streptomycin at 1 or 0.5 mg/mL and filter-sterilized with a 0.22 μm filter.

The cells were grown at 37 °C in a suitable incubator in a humidified atmosphere of 5% CO_2_ and routinely subcultured when reaching 70–80% confluency using 0.25% (w/v) trypsin–0.53 mM EDTA solution and reseeded at a subcultivation ratio of 1:5. The medium was renewed 1 to 2 times per week.

##### 2.7.3.2 MTT Assay

Cell viability was assessed using the 3-(4,5-dimethylthiazol-2-yl)-2,5-diphenyl-2*H*-tetrazolium bromide (MTT) assay, modified from Liu *et al*.^43^ A549 cells were seeded in a 96-well plate at 5000 cells/well 24 h before the experiment. Cells were then treated with 0.5 or 1.0 mg/mL methacrylated L-PEI, crotonylated L-PEI, maleylated L-PEI, and succinylated L-PEI dissolved in complete medium 1% FBS for 24 h. Cells treated with complete medium 1% FBS were used as a negative control and designated as 100% cell viability. 0.5 mg/mL of the toxic b-PEI was used as a positive control. After 24 h, test reagents were removed, and cells were washed with Hanks’ Balanced Salt Solution (HBSS) and incubated with 25 μL of MTT solution (5 mg/mL in HBSS) at 37 °C for 3 h to allow MTT reduction. The reaction was terminated by adding 275 μL of DMSO per well. Absorbance values at 570 nm were determined with a microplate reader by SpectraMaxi3x imaging cytometer Softmax Pro 7.2 (Molecular Devices, US), using 630 nm as the reference wavelength. The results are given as cell viability (%) relative to the negative control (1% FBS) and were calculated using the following equation:
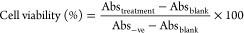
7where Abs is absorbance and Abs_–ve_ is negative control (=complete medium 1% FBS).

All values are reported as the mean ± standard deviation of a total of six biological replicates.

#### 2.7.4 Measurement of Cell Death

Cell death was evaluated using 6-diamidino-2-phenylindole (DAPI) and propidium iodide (PI) staining.^44^ A549 cells were plated in 12-well plates (5 × 10^4^ cells/well) 24 h before the experiment.

The cells were then treated with aqueous solutions of methacrylated L-PEI or crotonylated L-PEI (0.5 or 1.0 mg/mL) or with complete medium 1% FBS (negative control) or with aqueous solutions of b-PEI (0.5 mg/mL) used as a positive control known to cause cell apoptosis. After 24-h treatment, cell monolayers were washed twice with 0.75 mL of Dulbecco’s phosphate-buffered saline (DPBS) and incubated with 0.75 mL of DAPI (100 μM) and PI (35 μg/mL) for 10 min. The cells were then washed for 30 min at 10-min intervals with 0.75 mL of DPBS under light-shielded conditions. The cells were fixed in 0.75 mL of 4% formaldehyde solution in the dark for 10 min at room temperature. The cells were then washed once with DPBS and observed using an Invitrogen EVOS FL Digital Inverted Fluorescence Microscope, under a 40× objective, with DAPI (360 nm excitation, 447 nm emission) and RFP (530 nm excitation, 593 nm emission) light cubes to visualize DAPI and PI staining, respectively. Fluorescence images were taken for each well using the EVOS FL Cell Imaging System Software. The cell-permeable DAPI stained all cells (*N*_DAPI_), whereas PI (*N*_PI_), normally an impermeant fluorescent dye, stained dead cells with impaired plasma membrane permeability. Cell mortality (%) was calculated using the following equation:
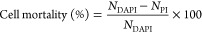
8

All values are reported as the mean ± standard deviation for a total of nine biological replicates.

### 2.8 Statistical Analysis

Student’s *t* test and one-way analysis of variance (ANOVA) were used to calculate *p* values, where *p* < 0.05 was set as the statistical significance criterion. The SMIT data were evaluated for significance using ANOVA followed by a Bonferroni’s post hoc test using GraphPad Prism software (version 8.0.2; GraphPad Software Inc., San Diego, CA, USA), where *p* < 0.05 was set as the statistical significance criterion.

## 3 Results and Discussion

### 3.1 Synthesis and Characterization of Methacrylated L-PEI, Crotonylated L-PEI, Maleylated L-PEI, and Succinylated L-PEI

**Scheme 1:**
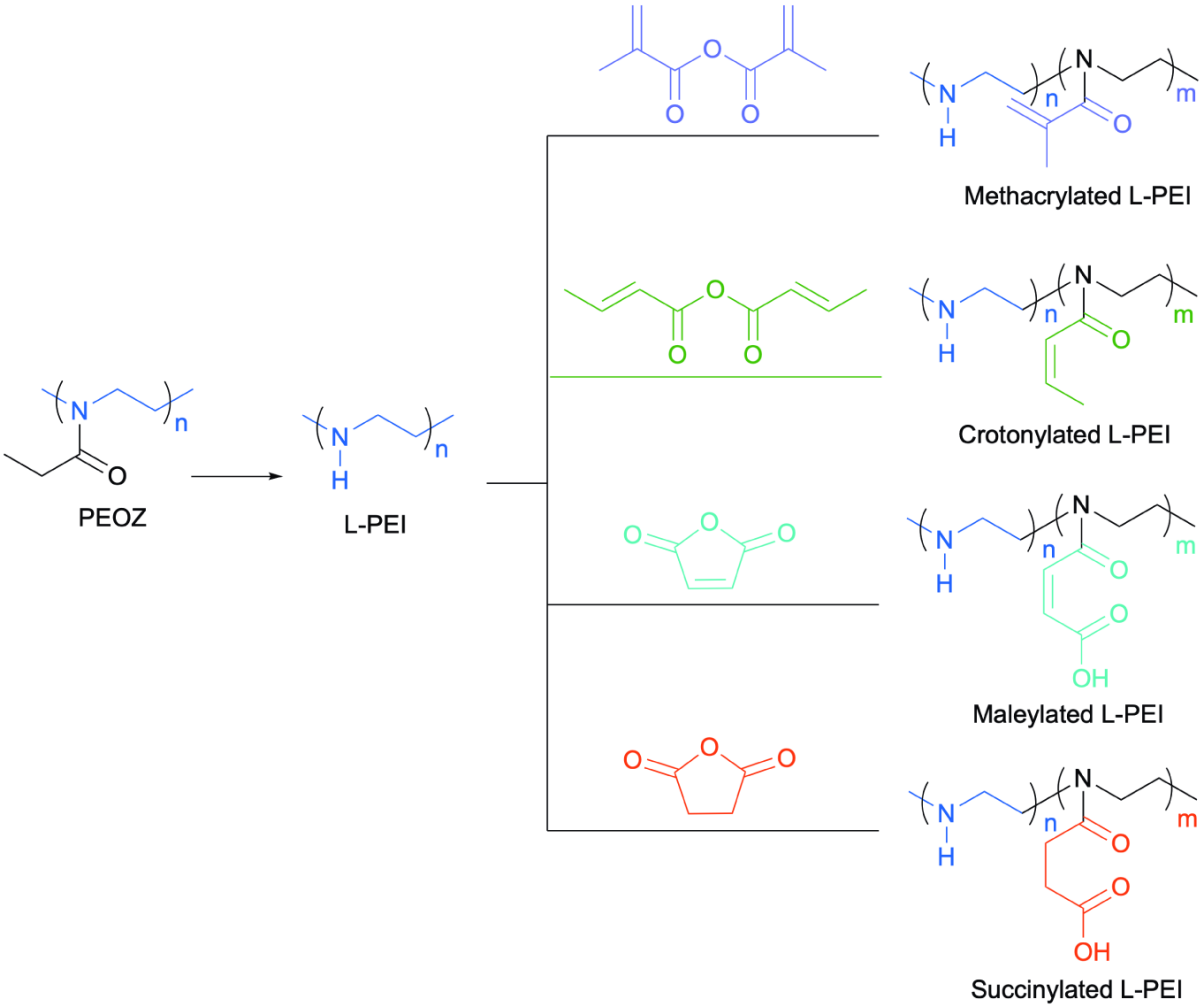
L-PEI was Obtained by Acidic Hydrolysis of PEOZ and Subsequently Modified with Methacrylic Anhydride, Crotonic Anhydride, Maleic Anhydride, and Succinic Anhydride, Respectively

**Figure 1:**
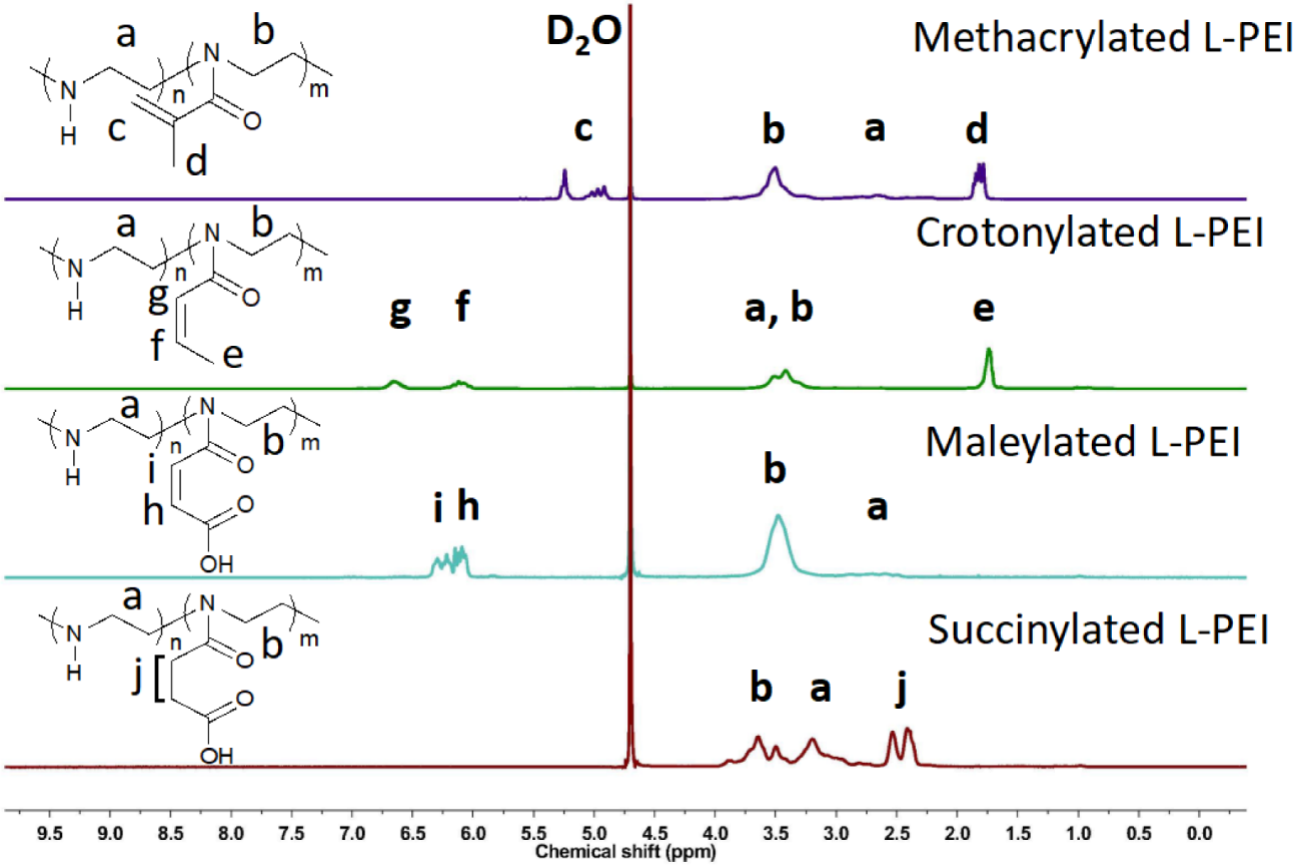
^1^H NMR spectra of methacrylated L-PEI, crotonylated L-PEI, maleylated L-PEI, and succinylated L-PEI recorded in D_2_O.

**Figure 2:**
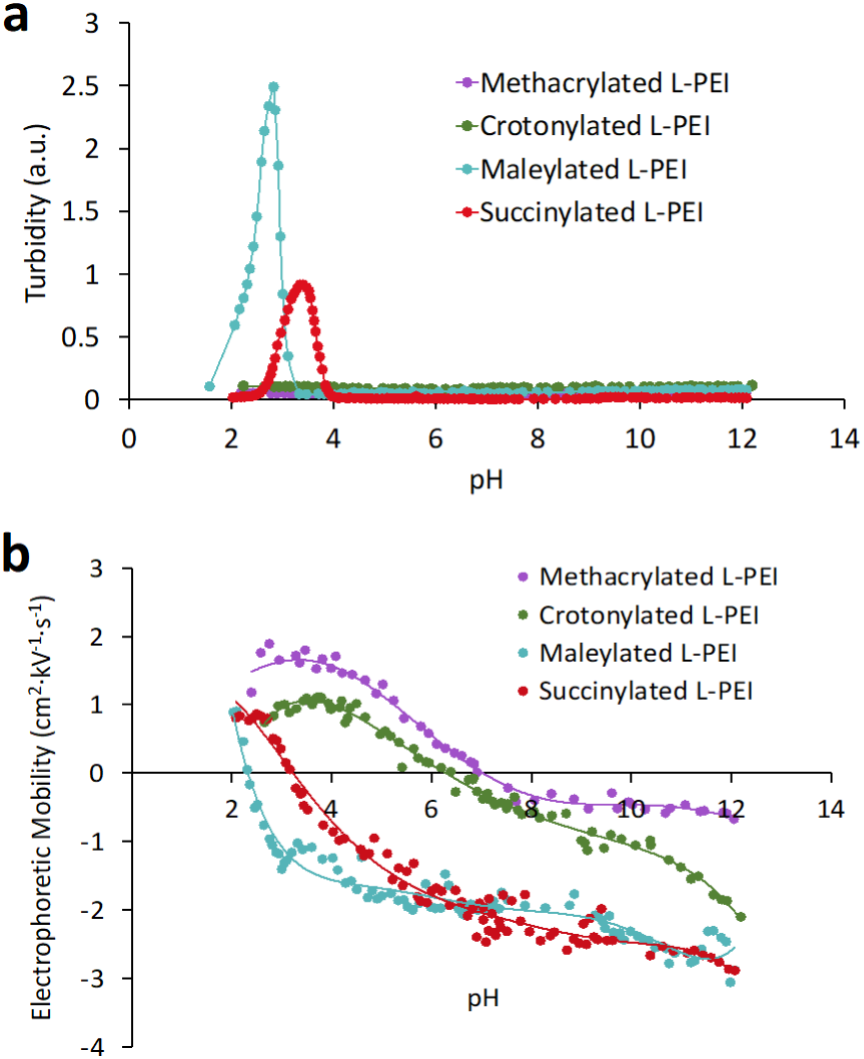
Effect of pH on solution turbidity (a) and electrophoretic mobility (b) of 1 mg/mL methacrylated L-PEI, crotonylated L-PEI, maleylated L-PEI, and succinylated L-PEI aqueous solutions.

Shan *et al*.^34^ previously reported a methodology to synthesize poly(2-oxazolines) from commercially available poly(2-ethyl-2-oxazoline) (PEOZ). Here, a similar strategy was used to synthesize methacrylated L-PEI, crotonylated L-PEI, maleylated L-PEI, and succinylated L-PEI from commercially available PEOZ (50 kDa). First, L-PEI was prepared via acidic hydrolysis of PEOZ^45^ (Scheme 1) with full conversion confirmed by ^1^H NMR (Figure 1S) and FTIR spectroscopies (Figure 2S). The main backbone signal of PEOZ and signals at 3.56, 1.12, and 2.44 ppm from its side chains disappeared from the NMR spectrum, but a signal typical for the L-PEI backbone was recorded at 2.75 ppm. Hydrolysis of PEOZ to form L-PEI was also confirmed by FTIR through the loss of the PEOZ amide carbonyl group at 1628 cm^–1^ and the presence of new strong bands at 1470 and 3259 cm^–1^, consistent with the N–H bend of L-PEI.^20^ The obtained L-PEI was reacylated via reaction with methacrylic anhydride, crotonic anhydride, maleic anhydride, and succinic anhydride in DMSO, with addition of triethylamine as a base. The resultant polymers were also characterized by ^1^H NMR and FTIR spectroscopies as well as using GPC.

As shown in Figure 1, the backbone −CH_2_–CH_2_– of L-PEI repeating units appeared at 2.75–3.50 ppm (signal a), which shifted to 2.90–3.75 ppm (signal b) upon acylation with the different anhydrides as new amide groups formed. For methacrylated L-PEI, signal d at 1.89 ppm and signal c at 5.08 ppm were assigned to −CH_2_– and −CH_3_ in the side group, respectively. For crotonylated L-PEI, signal e at 1.68 ppm, signal f at 6.05 ppm, and signal g at 6.63 ppm are attributed to the −CH-, −CH_2_–, and −CH_3_ in the side group, respectively. For maleylated L-PEI, signals h and i at 6.01–6.37 ppm were assigned to −CH_2_– in the side group. For succinylated L-PEI, signal j at 2.28–2.63 ppm corresponded to the −CH_2_– in the side group. The DS of methacrylated L-PEI, crotonylated L-PEI, maleylated L-PEI, and succinylated L-PEI were 83%, 93%, 80%, and 89%, as calculated using eqs 1 – 4, respectively.

**Figure 3:**
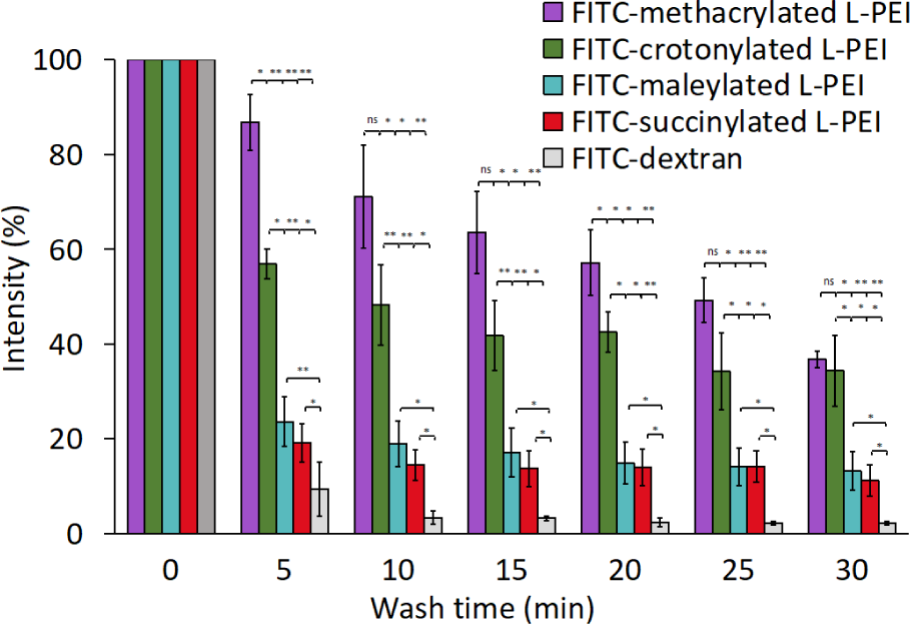
Retention of FITC-methacrylated L-PEI, FITC-crotonylated L-PEI, FITC-maleylated L-PEI, FITC-succinylated L-PEI, and FITC-dextran on bovine palpebral conjunctiva when washed with STF (0.1 mL/min) for 30 min at 34.5 ± 0.1 °C. Mean ± standard deviation, *n* = 3. The statistically significant differences are represented as **p < 0.01; *p < 0.05; ns, no significance.

Further confirmation of successful synthesis was provided by FTIR spectroscopy (Figure 3S and Table 1S). In particular, the FTIR spectra of the anhydride-modified polymers display a new stretching mode at 1611 cm^–1^ (maleylated), 1631 cm^–1^ (succinylated), 1644 cm^–1^ (methacrylated), and 1657 cm^–1^ (crotonylated), which was attributed to the formation of an amide group. The peaks at 1563 and 1606 cm^–1^ were assigned to C=C stretching vibrations of maleic anhydride and crotonic anhydride residues, respectively. Additionally, a new feature at 1718 cm^–1^ was assigned to the = C–H stretch of the methacrylic anhydride residue following its modification of L-PEI. Further, new peaks from maleylated L-PEI and succinylated L-PEI at 1706 and 1709 cm^–1^, respectively, were attributed to the C=O stretch of the carboxylic acid groups in the side chain. The bands at 3356 and 3363 cm^–1^ correspond to the carboxyl group (O–H stretch) of succinylated L-PEI and maleylated L-PEI, respectively.

**Figure 4:**
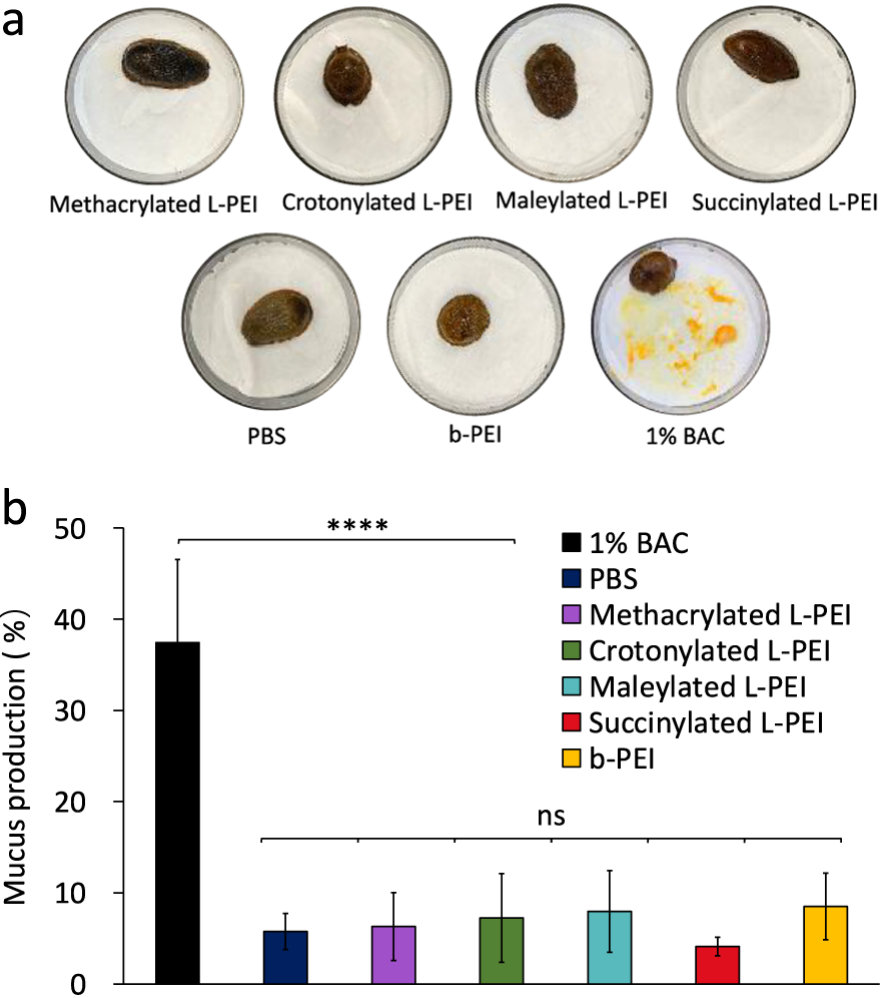
Exemplar images of slugs following 60 min of exposure to controls and test solutions using a slug mucosal irritation test (a); and mucus production in contact with 1% BAC in PBS, PBS, methacrylated L-PEI, crotonylated L-PEI, maleylated L-PEI, succinylated L-PEI, and branched PEI (b). Data are given as mean ± standard deviation (*n* = 5). Statistically significant differences were represented as ****p < 0.0001; ns, no significance.

The parent PEOZ, along with selected samples of methacrylated and crotonylated L-PEI, were additionally analyzed using GPC. The results are presented in Table 2S and Figure 4S. Unfortunately, it was not possible to record the chromatogram for L-PEI, most likely due to its precipitation from the DMF once cooled. The molecular weight of PEOZ was determined to be lower (MW = 24.8 kDa) than that stated in the technical data provided by the manufacturer. The methacrylated and crotonylated L-PEI samples exhibited molecular weights of 19.4 and 19.7 kDa, respectively. These results are consistent with the chemical transformations conducted on PEOZ and subsequently on L-PEI. No cross-linking of the samples was observed.

The effects of pH on the turbidity and electrophoretic mobility of the anhydride-modified polymers in solutions were studied, with results summarized in Figure 2.

Turbidity–pH and electrophoretic mobility–pH profiles for the maleylated and succinylated L-PEI are typical for polyampholytes, showing minimum solubility or net charge when pH = pH_IEP_ (isoelectric point).^46^ A reduction or increase in pH of polymer aqueous solutions (1 mg/mL) was achieved by the addition of small portions of 0.1 M NaOH or HCl. The turbidimetric technique gave pH_IEP_ for maleylated L-PEI of 2.81 ± 0.07 and for succinylated L-PEI of 3.41 ± 0.08. The solutions remained transparent until the pH approached the pH_IEP_ with a further pH rise, resulting in a dramatic increase in turbidity, reaching the maximum turbidity at pH = pH_IEP_. Subsequent addition of 0.1 M NaOH led the solution to become transparent again. When pH < pH_IEP_ or pH > pH_IEP_, the polymers provide excess positively or negatively charged groups and so are water-soluble, whereas when pH = pH_IEP_, the polymer carries a net neutral charge and loses its solubility. The unsaturated maleic acid residue present in maleylated L-PEI may have stronger electron-withdrawing ability, which facilitates dissociation of the carboxyl group; this in turn may be a reason for a lower pH_IEP_ compared to the value recorded in the case of saturated succinylated L-PEI. Since methacrylated L-PEI and crotonylated L-PEI are not polyampholytes, they do not exhibit pH-dependent solubility behavior (i.e., they do not display the presence of the isoelectric point).

Electrophoretic mobility measurements are also suitable to determine the isoelectric point in polyampholytes.^47,48^ The electrophoretic mobility measurements gave pH_IEP_ of 2.30 ± 0.07 for maleylated L-PEI and of 3.16 ± 0.09 for succinylated L-PEI, which was slightly different from the pH_IEP_ values determined using turbidity–pH measurements. This could be attributed to the different principles of the measurements; the turbidity–pH measurements are based on aggregation of polymers at pH_IEP_, whereas the EM–pH measurements record the migration of particles to an oppositely charged electrode in an electric field. Although crotonylated L-PEI and methacrylated L-PEI are not polyampholytes, they still show charge reversion at pHs 6.51 ± 0.14 and 7.05 ± 0.15, respectively, which may be explained by the presence of counterions surrounding each macromolecular coil or particles and changes in their net charge. The DS of methacrylated L-PEI was lower than the DS value for crotonylated L-PEI, resulting in fewer −NH– groups present in the later derivative. More −NH– groups available for protonation will result in greater pH_IEP_ values.

### 3.2 *Ex Vivo* Mucoadhesion Studies of Methacrylated L-PEI, Crotonylated L-PEI, Maleylated L-PEI, and Succinylated L-PEI

Mucoadhesive properties of the synthesized polymers were investigated using a fluorescence flow through method.^49^ Ocular mucosa was selected to evaluate mucoadhesive properties of new polymers, as there is a strong need to develop new formulations with enhanced retention ability on these mucosal surfaces. The conjunctiva and cornea are the major barriers in ocular drug delivery.^50^ Ramsay *et al*.^50^ demonstrated that the cornea provides near 10-fold greater barrier to drug permeation than the conjunctiva. Similarly, rabbit cornea was impermeable to FITC-dextran (MW 20 kDa), whereas it was able to permeate through the conjunctiva.^51^ Moreover, the conjunctiva has been reported to have permeability toward hydrophilic drugs than the cornea.^52^ The conjunctiva is a thin transparent membrane and covers the posterior surface of the upper and lower lids (palpebral conjunctiva) and the region from the upper and lower fornix over the sclera up to the cornea (bulbar conjunctiva).^53^ This layer contains goblet cells which are responsible for secreting mucins,^54,55^ and so may be a significant site for mucoadhesion to the ocular surface.^56^

**Figure 5:**
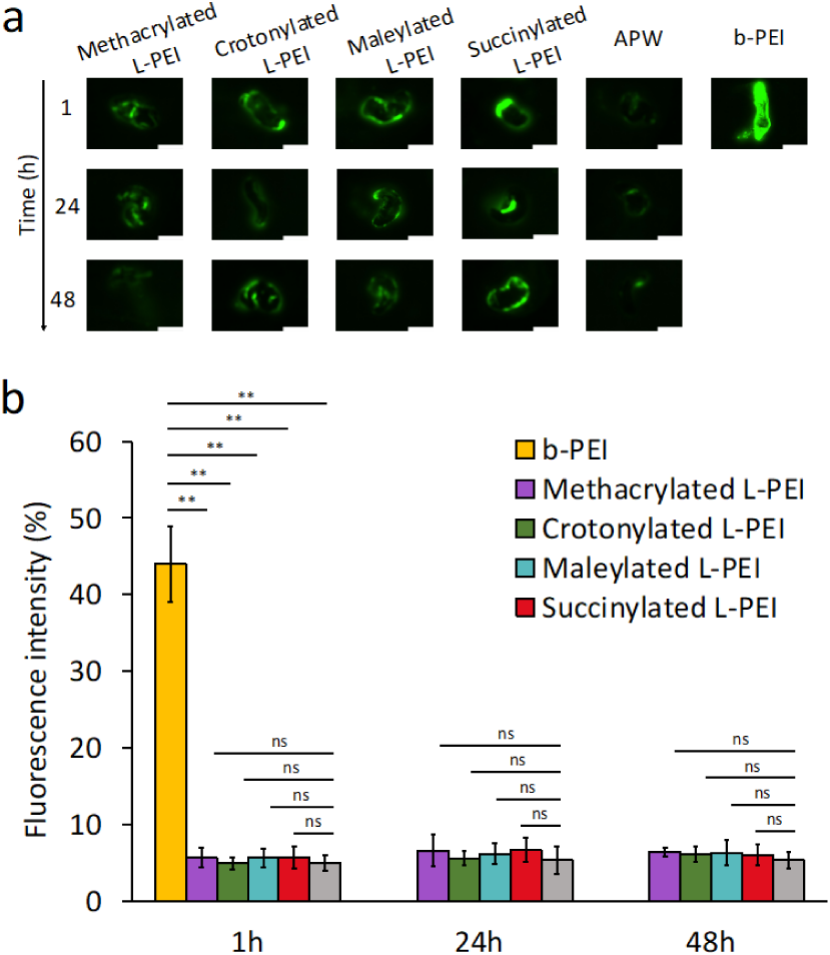
(a) Exemplar fluorescent images of individual planaria exposed to 1 mg/mL methacrylated L-PEI, crotonylated L-PEI, maleylated L-PEI, and succinylated L-PEI, with APW used as a negative control and 0.1 mg/mL b-PEI as a positive control. Note that fluorescent images could not be obtained after 24 and 48 h exposure to b-PEI as these conditions resulted in partial disintegration of the worms. Scale bar is 2 mm. (b) Mean fluorescence intensity values of planaria exposed to 1 mg/mL methacrylated L-PEI, crotonylated L-PEI, maleylated L-PEI, and succinylated L-PEI, with APW as negative control and 0.1 mg/mL b-PEI as positive control, calculated from the analysis of images. Data are given as the mean ± standard deviation (*n* = 5). The statistically significant differences are represented as **p < 0.01; ns, no significance.

**Figure 6:**
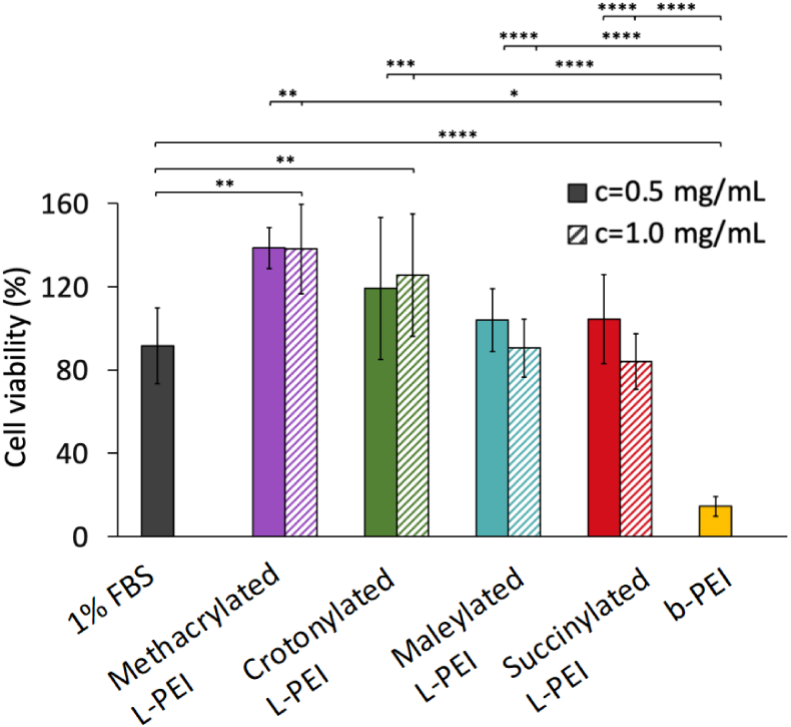
Viability of A549 cells determined after treatment with solutions of methacrylated L-PEI, crotonylated L-PEI, maleylated L-PEI, and succinylated L-PEI for 24 h using MTT assay. Cells treated with complete medium 1% FBS were used as a negative control, and cells exposed to 0.5 mg/mL b-PEI were used as a positive control. Data are expressed as % of external control, cells untreated, left in complete medium 10% FBS. Values are shown as mean ± standard deviation (*n* = 6 replicated per treatment). Statistically significant differences are represented as *p < 0.05; **p < 0.01; ns, ***p < 0.001; ****p < 0.0001.

First, methacrylated L-PEI, crotonylated L-PEI, maleylated L-PEI, and succinylated L-PEI were successfully labeled with FITC (Figure 5S). Retention of FITC-labeled compounds was evaluated on bovine palpebral conjunctiva washed with simulated tear fluid (STF) at pH = 7.4, with FITC-labeled dextran used as a negative control due to its well-documented poor mucoadhesive properties. The exemplar fluorescence images are shown in Figure 6S. All images were analyzed using ImageJ software (Figure 3).

Throughout 30 min of washing with STF, there was a statistically significant greater retention of all the studied L-PEI derivatives compared to the nonadhesive FITC-dextran. After 5-min washing, the retention of FITC-methacrylated L-PEI, FITC-crotonylated L-PEI, FITC-maleylated L-PEI, and FITC-succinylated L-PEI was 87%, 57%, 24%, and 18%, respectively, while the retention of FITC-dextran was 9%. The order of relative retention was maintained throughout the washout period, with 37%, 34%, 13%, and 11% of FITC-methacrylated L-PEI, FITC-crotonylated L-PEI, FITC-maleylated L-PEI, and FITC-succinylated L-PEI, respectively, remaining on the tissue after 30 min of washing. Although poorly mucoadhesive, 2% of FITC-dextran fluorescence remained after 30 min of washing, but this could be attributed to its penetration into the bovine conjunctiva tissue rather than adhesion to the surface.

The strong adhesion of FITC-methacrylated L-PEI and crotonylated L-PEI can be attributed to the presence of unsaturated C=C within these polymers, which could potentially form covalent bonds with thiol groups present on mucosal surfaces.^28^ The contribution of the amine groups within these polymers to adhesion will be minimal at pH to 7.4, since their macromolecules will be either noncharged or negatively charged. The methacrylated L-PEI displayed greater retention values than the crotonylated derivative possibly due to better tendency of methacryloyl groups to form covalent bonds with thiols compared to crotonyl groups, related to the steric hindrance of the methyl group.^57^

As described above, the pH_IEP_ of maleylated L-PEI and succinylated L-PEI, measured via the turbidimetric technique, was below pH 7.4, and so both of these polymers carry a net negative charge throughout this retention study. As both the polyampholytes and mucin carry a net negative charge, electrostatic interactions with mucosal surface are unlikely. Other mucoadhesive mechanisms may operate such as interdiffusion when polymers are in intimate contact with the mucus layer.^58,59^ It is likely that the polyampholytes penetrated into the bovine palpebral conjunctiva tissue synergized with diffusion of soluble mucins from the tissue, as has been previously reported.^60^ In general, the diffusion of macromolecules into the mucus gel is significantly influenced by factors such as molecular weight, charge, the presence of specific functional groups, and chain flexibility. Macromolecules that are smaller, more flexible, and less charged in nature are expected to exhibit a greater diffusivity into the mucus gel. FITC-maleylated L-PEI showed greater retention than FITC-succinylated L-PEI, perhaps due to the presence of the C=C bond, potentially capable of forming covalent bonds with thiol groups in mucin, which is absent in the succinylated polymer. It should also be noted that maleyl groups, likely due to their lower reactivity with thiols, exhibit weaker capacity to enhance mucoadhesive properties compared with methacryloyl and crotonyl groups. It is also evident that all polymers were retained to a greater extent than FITC-dextran; again, it is likely that our linear and flexible polymers diffuse into the mucus layer more readily than dextran.

### 3.3 Slug Mucosal Irritation Test

A slug mucosal irritation test was developed by Adriaens *et al*.,^61,62^ measuring slug mucus production (MP%) to evaluate the irritation potential of pharmaceutical compositions on mucosal surfaces. Here, a modified version of the test previously developed within our research group was used to assess irritation of methacrylated L-PEI, crotonylated L-PEI, maleylated L-PEI, and succinylated L-PEI,^63,64^ with PBS as a negative control and 1% benzalkonium chloride (BAC) and b-PEI as two positive controls. Since aqueous solutions of L-PEI tend to form physical gels,^65^ it was unsuitable for use as a control in these experiments. Therefore, b-PEI was selected as an additional positive control due to its well-documented toxicity, good aqueous solubility, and structural similarity to our polymeric derivatives.

Figure 4 gives exemplary images of slugs after 60 min of exposure to 1% BAC in PBS solution (positive control), PBS solution (negative control), and 1.0 mg/mL methacrylated L-PEI, crotonylated L-PEI, maleylated L-PEI, succinylated L-PEI, and b-PEI solutions, along with mucus production values. A significant irritation response was evident in slugs exposed to 1% BAC, reaching 37 ± 9% mucus production. It should be noted that these positive control results have significant variability due to the slugs’ increased activity and movement to minimize contact with the irritant, but our data are in accord with prior reports.^36,64^ As expected, exposure to the negative control (PBS) generated 6 ± 2% of mucus production, consistent with previous reports by Adriaens *et al*.^61,62^ and by Khutoryanskaya *et al*.^63^ Mucus production following exposure to our mucoadhesive polymers was not statistically different from the amount of mucus produced in control slugs, treated with PBS (mucus production: methacrylated L-PEI (6 ± 4%), crotonylated L-PEI (7 ± 5%), maleylated L-PEI (8 ± 4%), succinylated L-PEI (4 ± 1%), and b-PEI solutions (8 ± 4%)). These results suggest that the novel polymers are not strong irritants though it should be noted that b-PEI solutions also did not show significant mucosal irritation in slugs whereas it has well documented toxic properties in various cell culture assays.^66−68^

### 3.4 Toxicological Tests in Live Planaria

**Figure 7:**
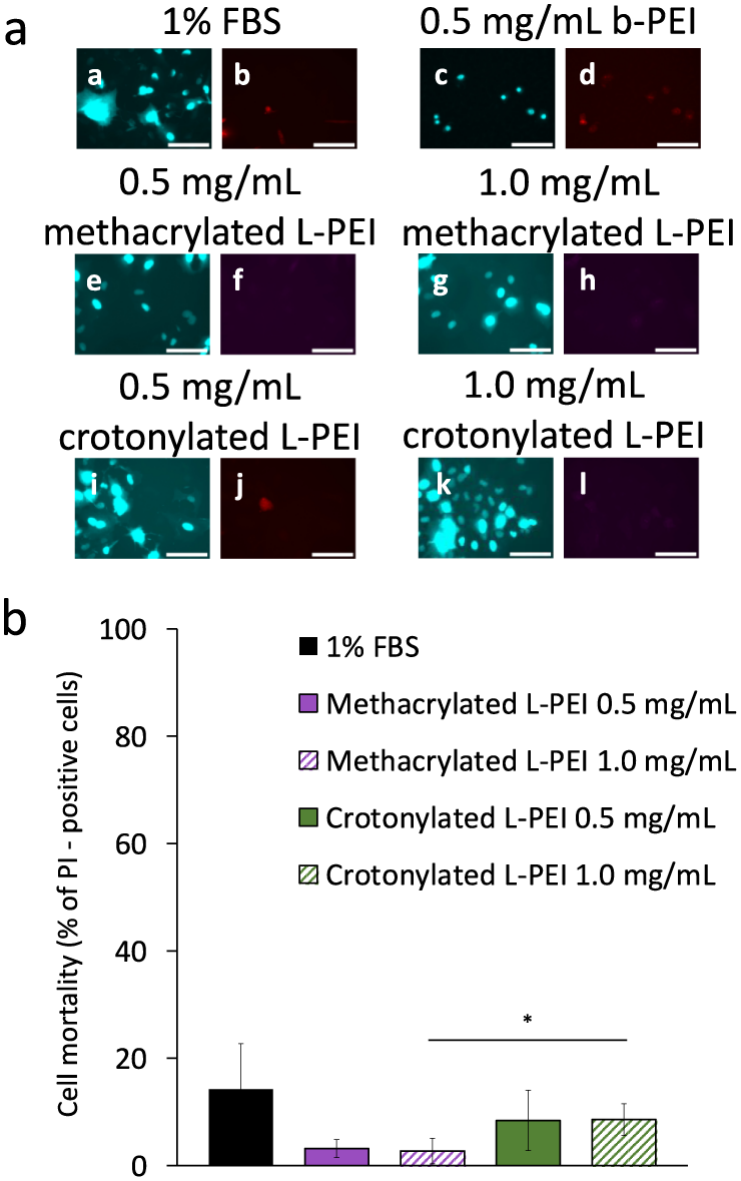
Mortality of A549 cells evaluated after treatment with methacrylated L-PEI and crotonylated L-PEI at 0.5 and 1.0 mg/mL for 24 h, with untreated cells in 1% FBS as the negative control (a); representative DAPI (left) and PI (right) staining images of methacrylated L-PEI and crotonylated L-PEI at 0.5 and 1.0 mg/mL, with cells cultured in 1% FBS as a negative control and cells exposed in 0.5 mg/mL b-PEI as a positive control, scale bar is 100 nm (b). Cell mortality % is expressed as values are expressed as means ± standard deviation (*n* = 3). Statistically significant differences are represented as *p < 0.05.

Planaria are aquatic flatworms that have been recently proposed by our research group as an *in vivo* model for screening irritancy potential of formulations.^32^ In this study, the potential toxicity of the novel polymers was evaluated using two *in vivo* assays in planaria.^32,42^ In the acute toxicity assay, live planaria were exposed to 1 mg/mL polymer solutions for up to 48 h. Under these conditions, all planaria survived throughout exposure, similar to worms that were exposed to PBS and artificial pond water, used as two negative controls. Planaria exposed to b-PEI at one tenth the above concentration (0.1 mg/mL) only survived for up to 1 h, before partially disintegrating at 24 and 48 h (Figure 7S). It should be noted that this test cannot be performed using L-PEI due to its tendency to form gels.^65^ The toxic nature of b-PEI is well documented in cell cultures,^68−70^ and so was expected to have toxic effects on planaria. The results of this study indicate that chemical modification of L-PEI with anhydrides results in polymeric derivatives that reduce the toxicity of the parent material.

The effects of the new polymers on the integrity of planaria epithelial membranes were explored using a fluorescence assay; Shah *et al*. demonstrated that sodium fluorescein can penetrate into planaria when its outer membrane is damaged following contact with irritant chemicals.^32^

Planaria initially exposed to polymer solutions for 1, 24, or 48 h, and subsequently exposed to solutions of sodium fluorescein, showed fluorescence levels similar to the negative controls or artificial pond water (Figure 5).

These results indicate that the synthesized polymers do not adversely affect the planarian membrane and were equivalent to the results following exposure to artificial pond water. However, there was a statistically significant increase in fluorescence intensity when planaria were exposed to the strongly irritant 0.1 mg/mL b-PEI, used at a tenth of the strength of our new materials. Though 0.1 mg/mL b-PEI was nonirritant to slugs, it showed toxicity to planaria, which may be explained by the ability of slugs to secrete a mucus layer that acts as a barrier to b-PEI, or simply due to differences in the resilience of the slug membrane compared to the more fragile and simpler planaria membrane.

The toxicity of the newly synthesized polymers was also investigated in human A459 epithelial cells using the MTT assay to measure cell viability. A549 cells have been tested in a variety of applications, as they model the alveolar Type II pulmonary epithelium and manufacture constructs for use in clinical trials. A549 cells are adenocarcinoma human alveolar basal epithelial cells, which have been extensively applied in toxicology, drug therapy, and pharmacological studies.^71,72^

Figure 6 shows that all the new polymers at both concentrations (0.5 or 1.0 mg/mL) tested for 24 h did not alter the viability of the A549 cells when compared to complete medium 1% FBS as negative control. On the contrary, the toxic b-PEI^73,74^ (used as a positive control) significantly reduced the cell viability by 86%.

Interestingly, viable cell numbers increased when treated with methacrylated L-PEI by 47% at both concentration and with crotonylated L-PEI treatment increased 28% (at 0.5 mg/mL) and 34% (at 1.0 mg/mL) compared to complete medium 1% FBS (control). It is feasible that these polymers promote cell growth and proliferation, as reported previously for a methacrylic anhydride-modified gelatin hydrogel.^75^

To further confirm the safety of our polymers, we assessed the plasma membrane integrity following 24 h treatment, using the normally impermeant fluorescent DNA-binding dye PI^44^ to stain the DNA of dead cells,^76^ used in tandem with the nucleic acid stain DAPI, used to determine both cell numbers and thus proliferation. Also in this case, complete medium 1% FBS and 0.5 mg/mL b-PEI were used as negative and positive control, respectively.

Figure 7 illustrates cell mortality following treatment with polymer solutions. Cell mortality from methacrylated L-PEI at 0.5 and 1.0 mg/mL was 3.2% and 2.7%, respectively, while mortality from crotonylated L-PEI at 0.5 and 1.0 mg/mL was 8.4% and 8.6%, respectively; both values are below that of cells cultured in 1% FBS (14.2%) while mortality following b-PEI treatment was 100% (*n* = 6). These results confirm the earlier findings that the new polymers show no adverse effects on cell viability and indeed suggest that they may have some protective effects against cell death.

## 4 Conclusion

In this work, cationic and amphoteric mucoadhesive polymers were synthesized by modification of L-PEI with methacrylic anhydride, crotonic anhydride, maleic anhydride, and succinic anhydride. Successful modification of L-PEI was confirmed using ^1^H NMR and FTIR spectroscopies, with the formation of derivatives containing 83%, 93%, 80%, and 89% of methacrylated, crotonylated, maleylated, and succinylated groups, respectively. The mucoadhesive properties and mechanisms of action at physiological pH (7.4) were explored using a fluorescence flow through method on bovine palpebral conjunctiva tissue. Methacrylated L-PEI and crotonylated L-PEI showed greater mucoadhesion than the two amphoteric polymers due to their ability to form covalent bonds with thiols present on mucosal surfaces. L-PEI modified with maleyl groups exhibited a weaker capacity to enhance mucoadhesive properties compared to the polymer derivatives containing methacryloyl and crotonyl groups. This is likely to be related to a weaker ability of maleyl groups to form covalent bonds with thiols compared to methacryloyl and crotonyl groups. The toxicological properties of modified L-PEI materials were assessed using an MTT assay, slug mucosal irritation assay, and planaria-based assays. Irritation studies conducted on slugs showed no evidence that the new materials were irritants to mucosal membranes. The rapid and low cost planaria assay similarly demonstrated no significant damage to membranes at the concentrations employed. The MTT assay and DAPI/PI staining of A549 cells also demonstrated that the polymers had no appreciable toxicity in a human cell line. This work thus provides a series of anhydride-modified L-PEIs with improved biocompatibility and mucoadhesive properties that operate via a range of mechanisms from covalent bonding with mucins to electrostatic interactions or interdiffusion. The toxicological evaluation of b-PEI using slug mucosal irritation assay, planaria-based assays, and cell culture assay indicates that our new assays using planaria are more sensitive in detecting toxicity of compounds compared to the use of slugs.

## Supporting Information Available

^1^H NMR spectra of PEOZ and L-PEI recorded in methanol-d4; FTIR spectra of PEOZ and L-PEI; FTIR spectra of methacrylated L-PEI, crotonylated L-PEI, maleylated L-PEI, and succinylated L-PEI; fluorescence spectra of FITC-labeled methacrylated L-PEI, crotonylated L-PEI, maleylated L-PEI, and succinylated L-PEI at 1 mg/mL; exemplar images of ex vivo bovine palpebral conjunctiva with applied FITC-dextran, FITC-methacrylated L-PEI, FITC-crotonylated L-PEI, FITC-maleylated L-PEI, and FITC-succinylated L-PEI; acute toxicity assay was conducted after 48 h exposure of planaria to 1 mg/mL methacrylated L-PEI, crotonylated L-PEI, maleylated L-PEI, succinylated L-PEI, APW, and 24 h exposure of planaria to 0.1 mg/mL b-PEI; FTIR absorption bands from methacrylated L-PEI, crotonylated L-PEI, maleylated L-PEI, and succinylated L-PEI; GPC traces and molecular weight data for PEOZ and selected methacrylated and crotonoylated L-PEI derivatives (PDF)
