# Supplementary material for: Exploring Mucoadhesive and Toxicological Characteristics Following Modification of Linear Polyethylenimine with Various Anhydrides
Source: Biomacromolecules. 2024 Jul 29;25(8):4831–42. doi: 10.1021/acs.biomac.4c00220 (PMC11323015; doi:10.1021/acs.biomac.4c00220)
Supplement: Supplementary file 1 — bm4c00220_si_001.pdf [file bm4c00220_si_001.pdf]

Supporting Information for

Exploring Mucoadhesive and Toxicological Characteristics Following Modification of Linear Polyethyleneimine with Various Anhydrides

Manfei Fu[a], *Roman V. Moiseev*[a,b], *Matthew Hyder*[c], *Wayne Hayes*[c], *Silvia Amadesi*[a], *Adrian C. Williams*[a], *Vitaliy V. Khutoryanskiy**[a,b]

[a] School of Pharmacy, University of Reading, Whiteknights, Post Office Box 224, Reading RG6 6DX, United Kingdom

[b] Physicochemical, Ex Vivo and Invertebrate Tests and Analysis Centre (PEVITAC, www.pevitac.co.uk), University of Reading, Whiteknights, Reading, RG6 6DX, UK

[c]Department of Chemistry, University of Reading, Whiteknights, Post Office Box 224, Reading, RG6 6DX, UK

*Email: v.khutoryanskiy@reading.ac.uk

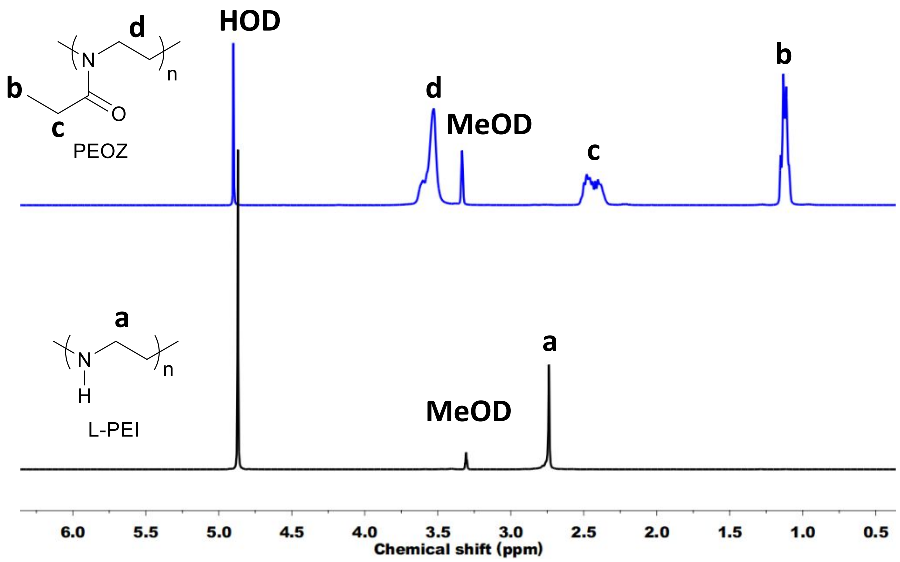

Figure 1S. ^{1}H-NMR spectra of PEOZ and L-PEI recorded in methanol-d4.

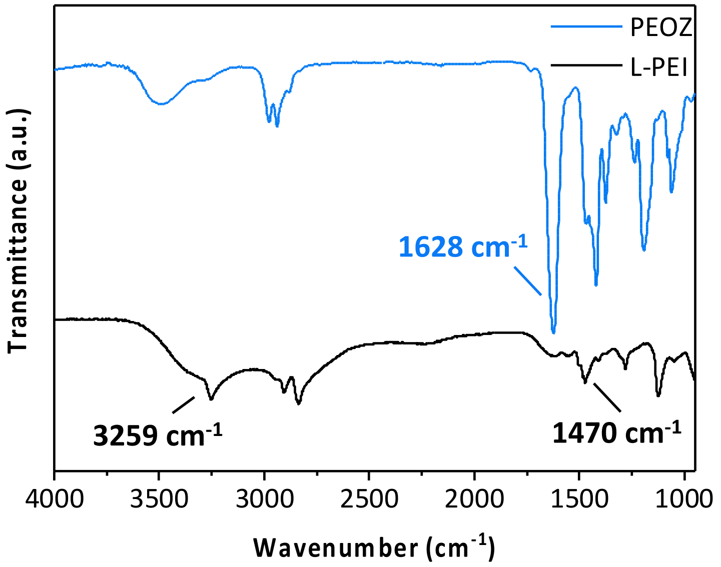

Figure 2S. FTIR spectra of PEOZ and L-PEI.

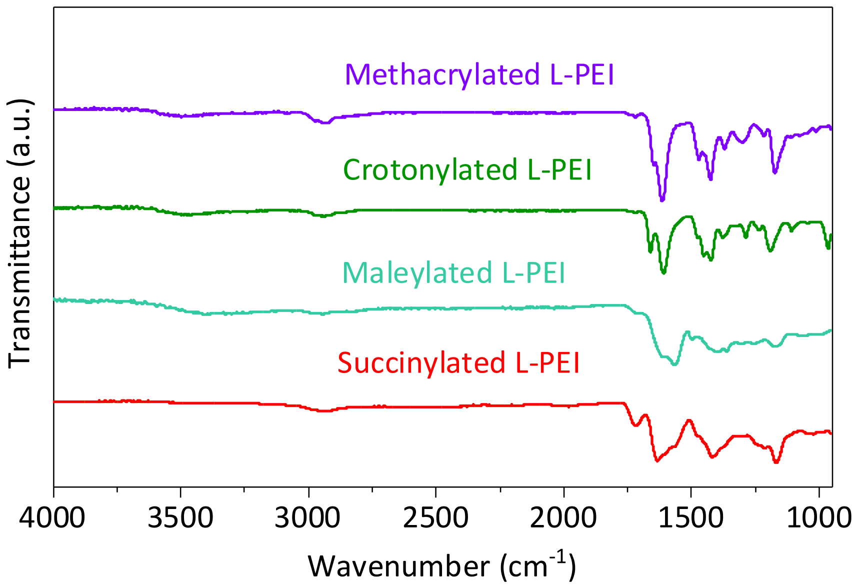

Figure 3S. FTIR spectra of methacrylated L-PEI, crotonylated L-PEI, maleylated L-PEI and succinylated L-PEI.

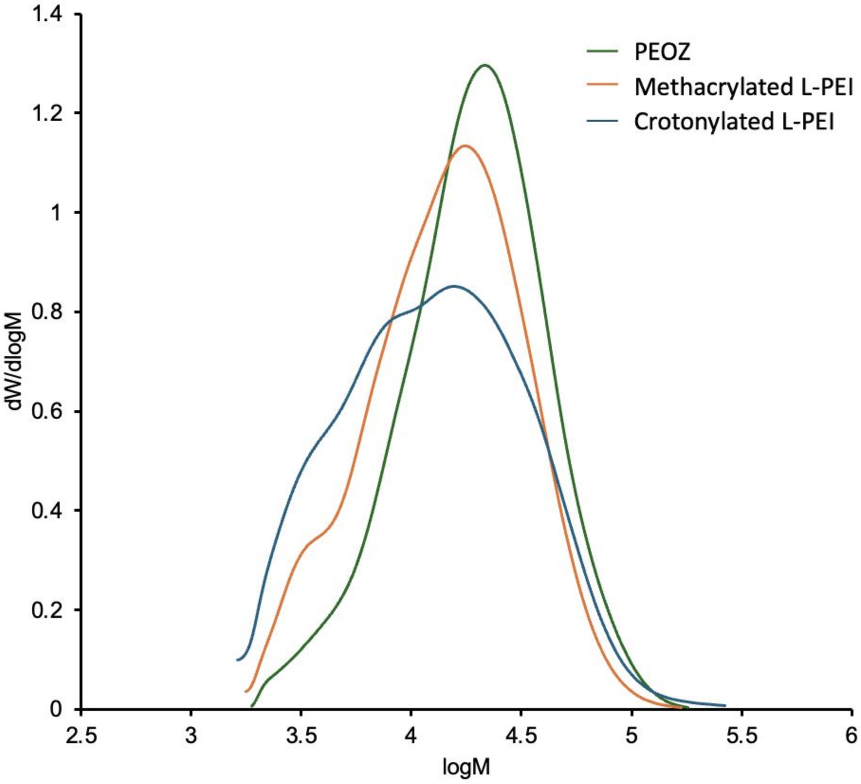

Figure 4S. GPC chromatogram of PEOZ, methacrylated L-PEI and crotonylated L-PEI in DMF.

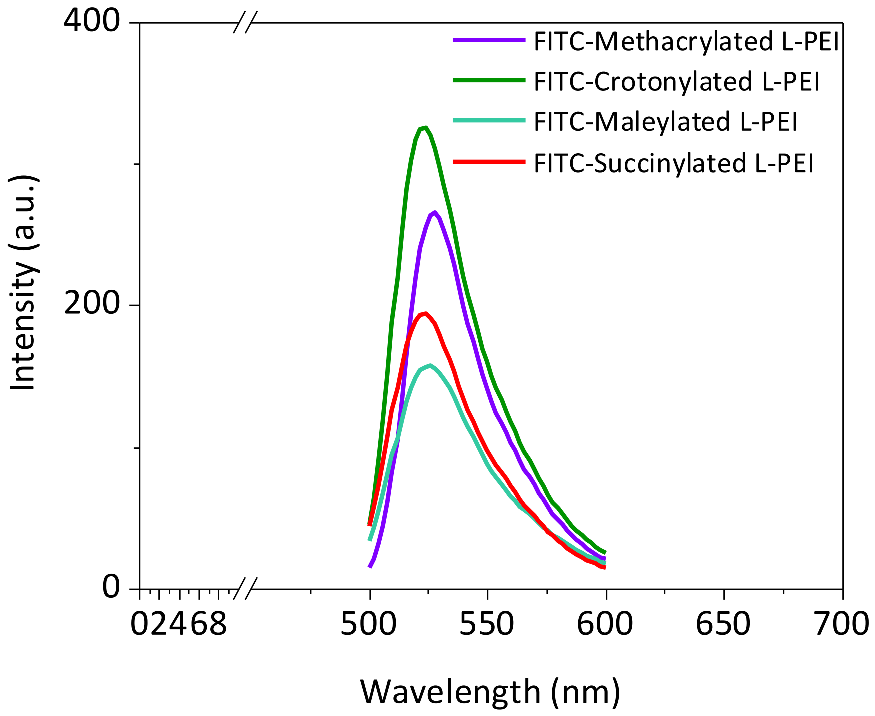

Figure 5S. Fluorescence spectra of FITC labelled methacrylated L-PEI, crotonylated L-PEI, maleylated L-PEI and succinylated L-PEI at 1mg/mL.

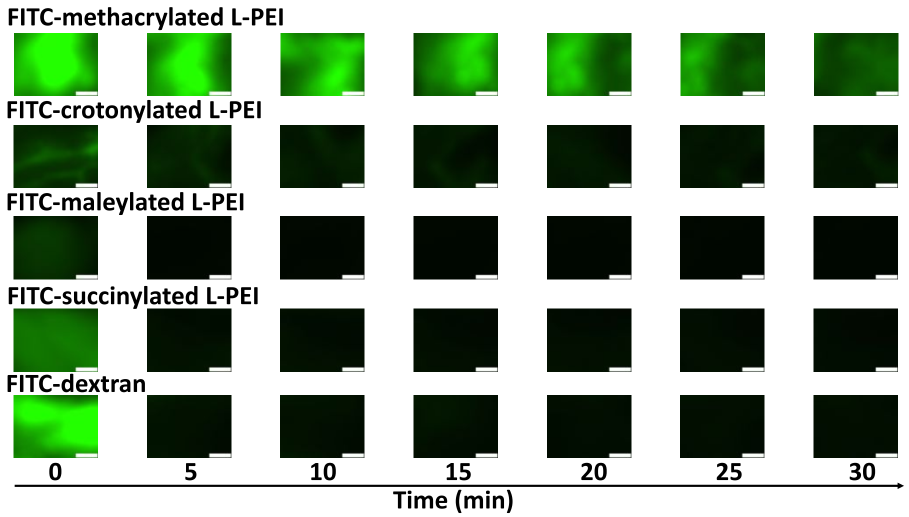

Figure 6S. Exemplar images of *ex vivo* bovine palpebral conjunctiva with applied FITC-dextran, FITC-methacrylated L-PEI, FITC-crotonylated L-PEI, FITC-maleylated L-PEI and FITC-succinylated L-PEI. Scale bars are 1 mm.

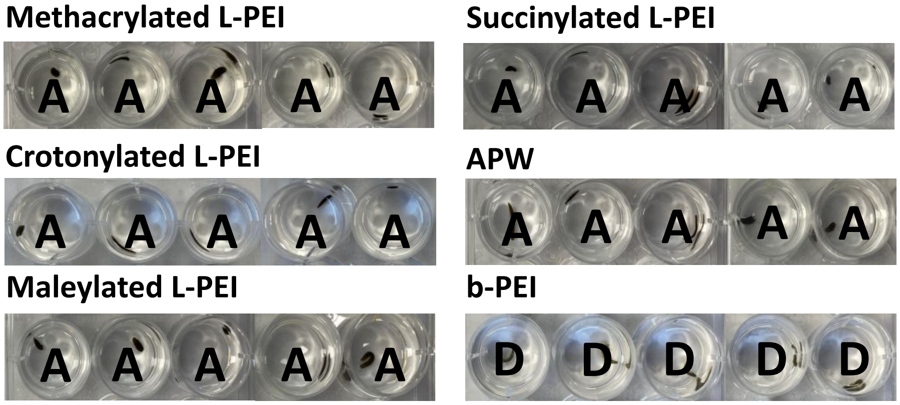

Figure 7S. Acute toxicity assay was conducted after 48h exposure of planaria to 1 mg/mL methacrylated L-PEI, crotonylated L-PEI, maleylated L-PEI, succinylated L-EPI, APW and 24h exposure of planaria to 0.1 mg/mL b-PEI. 'A' denotes live planaria whereas 'D' denotes dead planaria.

Table 1S. FTIR absorption bands from methacrylated L-PEI, crotonylated L-PEI, maleylated L-PEI and succinylated L-PEI

FTIR Absorption band of polymers (cm^{-1})				
Methacrylated L-PEI	Crotonylated L-PEI	Maleylated L-PEI	Succinylated L-PEI	Assignment
3495	3458	3466	3470	N-H stretch

/	/	3363	3356	O-H stretch
2938	2938	2927	2915	C-H stretch
1718	/	/	/	=C-H Stretch
1644	1657	1706， 1611	1709， 1631	C=O stretch
/	1606	1563	/	C=C stretch
1422	1420	1403	1416	C-H bend
1297	1285	1287	1299	C-N stretch
1171	1188	1173	1175	C-O stretch
974	962	988	988	C-H bend

Table 2S. Molecular weight and dispersity values of PEOZ and selected derivatives of L-PEI

Polymer	M_n (g mol^{-1})	M_w (g mol^{-1})	Đ
PEOZ	14100	24800	1.76
Methacrylated L-PEI	10200	19400	1.90
Crotonylated L-PEI	8300	19700	2.37
